# LGMD R18 Mimicking Wilson Disease: 6-Year Longitudinal Muscle MRI Evolution and Clinical Insights into *TRAPPC11* Mutation

**DOI:** 10.3390/diagnostics16111729

**Published:** 2026-06-04

**Authors:** Yen-Ting Chou, Hsiao-Ping Chou, Yu-Wei Chen

**Affiliations:** 1 Department of Pediatrics, Yonghe Cardinal Tien Hospital, New Taipei City 234, Taiwan; billyytc@gmail.com; 2 Department of Radiology, Yonghe Cardinal Tien Hospital, New Taipei City 234, Taiwan; gutmomo@gmail.com

**Keywords:** limb-girdle muscular dystrophy R18, Wilson disease, muscle MRI

## Abstract

**Background and Clinical Significance**: Limb-girdle muscular dystrophy R18 is a rare autosomal recessive disorder that may present with both muscular and hepatic involvement, potentially leading to diagnostic confusion. Longitudinal MRI data documenting this disease from childhood remain scarce. **Case Presentation**: We report a female patient with disease onset that occurred at 8 years of age, initially presenting with elevated liver enzymes and decreased ceruloplasmin levels, which raised suspicion of Wilson’s disease. Genetic testing confirmed TRAPPC11 mutation. Serial MRI scans of the thigh muscles over a six-year period demonstrated progressive fatty infiltration, predominantly affecting the adductor magnus and posterior thigh muscles, with relative sparing of the gracilis and anterior compartment muscles. **Conclusions**: This case highlights that limb-girdle muscular dystrophy R18 can clinically mimic Wilson disease. Longitudinal muscle MRI offers valuable diagnostic insights and reveals a characteristic pattern of muscle involvement.

## 1. Introduction

Limb-girdle muscular dystrophies (LGMDs) constitute a heterogeneous group of inherited neuromuscular disorders characterized by progressive weakness predominantly affecting the pelvic and shoulder girdle muscles. Recent advances in molecular genetics have led to the identification of numerous causative genes and the refinement of disease classification, highlighting the clinical and genetic diversity within this group [1,2,3].

*TRAPPC11*-related LGMD R18 (previously as LGMD 2S) is a rare autosomal recessive disorder resulting from mutations in the TRAPPC11 gene, which encodes a subunit of the trafficking protein particle (TRAPP) complex that plays a critical role in vesicular transport and Golgi apparatus function [4]. Accumulating evidence indicates that disorders associated with the *TRAPPC11* variant present a wide range of phenotypic manifestations, encompassing limb-girdle muscle weakness, elevated serum creatine kinase levels, and involvement beyond the muscular system, notably hepatic abnormalities [5,6].

While magnetic resonance imaging (MRI) findings in muscular dystrophies such as Duchenne muscular dystrophy (*DMD*) and Becker muscular dystrophy (*BMD*) are documented [7,8], there is a relative paucity of literature addressing MRI characteristic LGMDs, with specific reference to LGMD R18 [9,10].

Due to the rarity and heterogeneity of this disease, clinical data are scarce, diagnosis is challenging, and long-term follow-up data in the literature are lacking. Here, we report a pediatric patient with genetically confirmed LGMD R18 who initially presented with hepatic dysfunction and reduced ceruloplasmin levels, mimicking Wilson’s disease (WD). Serial muscle MRI over a 6-year period demonstrated a distinctive and progressive pattern of selective muscle involvement. This case highlights the diagnostic value of longitudinal muscle imaging and expands the clinical and radiological spectrum of the disease.

## 2. Case Report

A female patient, born to non-consanguineous parents with an unremarkable family history and normal neurodevelopmental milestones, presented at age 9 after incidental detection of elevated liver enzymes. Laboratory tests revealed persistently elevated serum transaminases, increased creatine kinase (CK) levels, and low ceruloplasmin, prompting an evaluation for Wilson’s disease. However, slit-lamp examination showed no Kayser–Fleischer (K-F) rings. At the initial clinical presentation at 9 years of age, neurological examination demonstrated intact cranial nerve function, normal and symmetric deep tendon reflexes, and preserved sensory function to all modalities. No extrapyramidal or cerebellar signs, including tremor, dystonia, bradykinesia, ataxia, or dysarthria, were observed. Muscle tone was normal, and Gowers’ sign was negative at that time. Although the patient subjectively reported reduced athletic performance and being the slowest runner in her class, objective examination initially revealed only subtle proximal muscle weakness without overt functional limitation. Testing for *ATP7B* variants associated with Wilson’s disease yielded negative results. Given the persistently elevated creatine kinase levels, an underlying neuromuscular disorder was suspected. Brain MRI revealed unremarkable intracranial structures, with no structural or focal signal abnormalities in the basal ganglia or brainstem. Needle electromyography (EMG) findings were consistent with a primary myopathic process. Comprehensive genetic analysis using a targeted next-generation sequencing panel for neuromuscular disorders subsequently identified a homozygous pathogenic variant in the *TRAPPC11* gene (c.2938G>A (p.G980R)), establishing the diagnosis of limb-girdle muscular dystrophy R18.

Serial laboratory evaluations over the six-year follow-up period demonstrated persistently elevated serum CK levels alongside consistently reduced ceruloplasmin levels. CK values showed notable fluctuations, ranging from 3901 to 10,147 IU/L (normal range: 45–163 IU/L), with a mean of 7429 IU/L and a standard deviation of 1615 IU/L, and remained markedly elevated throughout the observation period, consistent with ongoing muscle injury. In contrast, serum ceruloplasmin levels exhibited relatively mild fluctuations, ranging from 7.6 to 12.0 mg/dL (normal range: 17–31 mg/dL), with a mean of 10.1 mg/dL and a standard deviation of 1.3 mg/dL, but remained persistently below the normal reference range without a clear trend toward normalization.

**Figure 1:**
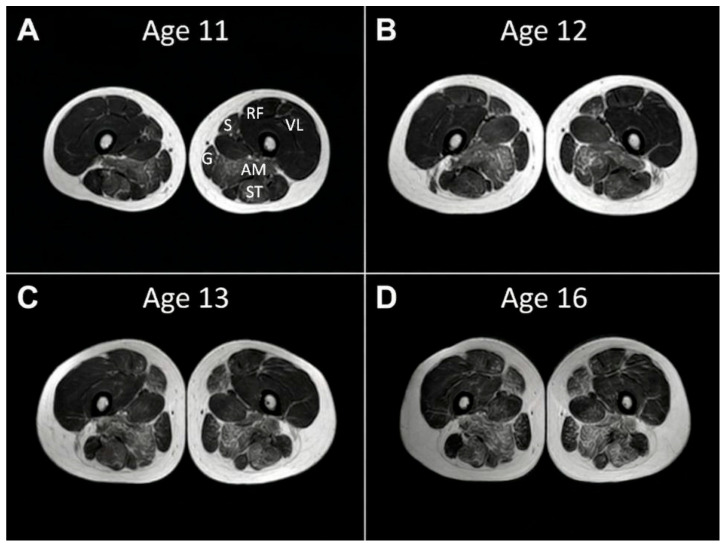
Longitudinal axial T2-weighted MRI of the thighs from age 11 to 16 years. Serial imaging demonstrates a progressive, symmetric fatty replacement with a distinctive “posterior-to-anterior gradient”. (**A**) Age 11: Early fatty infiltration (mild T2 hyperintensity) is confined to the posterior compartment, specifically the adductor magnus (AM) and semitendinosus (ST). (**B**) Age 12: Dystrophic changes progress in the posterior compartment, while the rectus femoris (RF), vastus lateralis (VL), sartorius (S), and gracilis (G) remain preserved. (**C**) Age 13: Accelerated fatty replacement is noted in the AM and hamstrings. Notably, the S and G begin to exhibit hyperintense streaking, marking the onset of involvement. (**D**) Age 16: Complete fatty replacement (Mercuri grade 4) involves the entire medial and posterior compartments (AM, ST, S, and G). In stark contrast, the anterior compartment (RF and VL) remains remarkably intact with preserved muscle volume and normal low T2 signal intensity.

Serial axial T2-weighted MRIs of the thighs were acquired at ages 11, 12, 13, and 16 years (Figure 1). Longitudinal evaluation demonstrated progressive and symmetric intramuscular fatty replacement, characterized by a distinct pattern of “posterior-to-anterior gradient”.

At baseline (age 11), mild T2 hyperintensity, indicative of early fatty infiltration, was predominantly confined to the posterior compartment, specifically localizing to the adductor magnus and semitendinosus muscles. By age 12, these dystrophic changes progressed, whereas the anterior compartment and superficial medial muscles (sartorius and gracilis) remained entirely preserved, retaining normal low T2 signal intensity. Follow-up imaging at age 13 revealed an accelerated expansion of T2 hyperintensity within the medial and posterior compartments, signifying advanced fatty replacement of the adductor magnus and hamstrings. Notably, the sartorius and gracilis, which were previously spared, began to exhibit distinct hyperintense streaking, marking the onset of their involvement. By age 16, complete fatty replacement (Mercuri grade 4) involved the entire medial and posterior compartments, encompassing the adductor magnus, hamstrings, sartorius, and gracilis. In stark contrast, the anterior compartment demonstrated marked relative preservation; the rectus femoris and vastus lateralis remained intact, maintaining normal muscle volume and low T2 signal. This sharp demarcation between the end-stage posterior-medial degeneration and the spared anterior musculature constitutes the radiological hallmark of this advanced phenotype.

As of her most recent follow-up at age 16, the patient is a senior high school student who remains independent in her activities of daily living (ADLs). She maintains a regular school schedule and an active social life, including shopping and traveling with her family. However, she experiences increasing difficulty and fatigue when climbing stairs due to progressive proximal muscle weakness. Consequently, she is unable to participate in competitive physical activities, such as running. The patient continues to receive regular functional assessments and clinical follow-up at our outpatient clinic. The patient’s detailed clinical course, longitudinal laboratory data, and the multi-step diagnostic trajectory over the 6-year follow-up period are chronologically summarized in Table 1.

## 3. Discussion

LGMD R18 represents a rare subtype of muscular dystrophy characterized by a heterogeneous clinical spectrum, encompassing progressive skeletal muscle degeneration and extra-muscular manifestations, notably hepatic involvement [1,6]. It has been established that LGMD can inadvertently mimic primary hepatic pathology. This phenomenon occurs because aminotransferases—specifically aspartate aminotransferase (AST) and alanine aminotransferase (ALT)—are highly concentrated within skeletal muscle tissue, not just the liver. Chronic sarcolemmal damage and myofiber degeneration in LGMD cause a massive release of these intracellular enzymes into the bloodstream. Clinically, AST levels initially rise more abruptly than ALT due to its higher intramuscular concentration; however, because of its shorter serum half-life, AST is cleared more rapidly. This temporal fluctuation often creates a biochemical profile that closely masquerades as acute or chronic liver injury, frequently leading to exhaustive gastrointestinal investigations and misdiagnoses [11]. Therefore, early screening of serum creatine kinase (CK) levels is vital to confirm a primary neuromuscular etiology rather than true hepatic injury, streamlining the diagnostic workflow toward targeted genetic testing.

The clinical presentation of our patient presented a diagnostic dilemma due to the coexistence of progressive myopathy, elevated liver enzymes, and persistently low serum ceruloplasmin levels—a biochemical profile closely mimicking WD. As highlighted in recent literature, disorders mimicking WD represent a growing diagnostic challenge, particularly when non-WD genetic or metabolic conditions exhibit overlapping hepatic and biochemical abnormalities [12]. Because WD is potentially treatable with early pharmacological agents, prompt and accurate differentiation is of paramount clinical importance [12]. In our patient, despite the initial suspicion raised by hypoceruloplasminemia, several pathognomonic features of WD were absent. Ophthalmologic slit-lamp examination demonstrated no Kayser–Fleischer rings, and brain MRI revealed no abnormalities typical of WD, such as basal ganglia or brainstem lesions. Furthermore, electromyography (EMG) demonstrated definitive myopathic changes consistent with a primary myogenic process, rather than the extrapyramidal or neurogenic involvement typically seen in neurologically symptomatic WD. Definitive genetic testing subsequently identified a homozygous pathogenic *TRAPPC11* variant, confirming the diagnosis of LGMD R18 and ruling out *ATP7B*-related disease.

Mechanistically, the decreased ceruloplasmin level in this patient likely reflects a secondary impairment of intracellular protein trafficking rather than primary copper toxicosis. Because the TRAPP complex is essential for endoplasmic reticulum-to-Golgi vesicular transport, *TRAPPC11* dysfunction may disrupt the secretory pathway and impair the processing or secretion of hepatocyte-derived glycoproteins like ceruloplasmin. Ultimately, this case underscores the necessity of including neuromuscular disorders in the differential diagnosis of pediatric patients with unexplained hepatic dysfunction and low ceruloplasmin. When these biochemical anomalies are accompanied by progressive skeletal myopathy, longitudinal muscle MRI profiling alongside next-generation sequencing is vital to prevent misdiagnosis and avoid unnecessary, lifelong copper-chelating therapy. To provide a clear clinical heuristic for distinguishing *TRAPPC11*-related myopathy from its metabolic phenocopies, a comprehensive comparative matrix was structured (Table 2).

A phenotypic comparison highlights a distinct clinical divergence between previously reported cases and our present case. The three individuals from a consanguineous Syrian family described by Bögershausen et al. [4] carried the homozygous c.2938G>A (p.Gly980Arg) variant in *TRAPPC11*. Similar to our patient, they presented with proximal muscle weakness and significantly elevated CK levels. However, their phenotypes exhibited extensive systemic and extra-muscular involvement, including skeletal abnormalities (hip dysplasia and scoliosis) in all patients, mild intellectual disability in one individual, and variable ocular manifestations. In contrast, our patient presents with a much milder, muscle-restricted phenotype. Following thorough clinical and radiological evaluations, our case demonstrated completely normal skeletal development and intact cognitive functions, with no signs of scoliosis, hip dysplasia, intellectual disability, or ocular involvement. This clinical divergence underlines a remarkable phenotypic heterogeneity associated with the homozygous *TRAPPC11* p.Gly980Arg variant, demonstrating that it can manifest either as a syndromic form of limb-girdle muscular dystrophy with skeletal and neurological symptoms, or as an isolated myopathy.

A primary strength of this case report is the six-year longitudinal MRI follow-up, which provides a unique opportunity to delineate the spatiotemporal progression of muscle involvement in TRAPPC11-related myopathy. Our longitudinal observations demonstrate that dystrophic changes in this condition do not occur uniformly but follow a highly selective trajectory. Specifically, fatty replacement was initially confined to the posterior compartment before extending to the medial compartment, whereas the anterior compartment remained relatively preserved even at advanced stages. This distinct “posterior-to-anterior gradient” serves as a critical radiological signature. To elucidate its diagnostic utility, it is instructive to compare these longitudinal findings with the classic imaging patterns of other muscular dystrophies.

### 3.1. Comparison with DMD/BMD

While DMD/BMD patients typically exhibit calf pseudohypertrophy, early-onset cardiomyopathy, and a relentless, non-fluctuating loss of ambulation milestones in early adolescence, our LGMD R18 patient demonstrated a more slowly progressive limb-girdle weakness, fully preserved ambulation at age 16, normal cardiac functions, and a prominent hypoceruloplasminemia never typical of pure dystrophinopathies.

The imaging trajectory in our patient reveals a distinct temporal divergence from the well-established hallmarks of *DMD* and *BMD*. At age 11, our patient demonstrated fatty replacement predominantly localized to the adductor magnus and semitendinosus. This early presentation shares a notable phenotypic overlap with *DMD/BMD*, which also frequently targets the posterior thigh early in the disease course [7,13]. In the absence of longitudinal data, this similarity could easily lead to diagnostic confusion. However, as the disease progressed, the imaging signatures markedly diverged. A classic radiologic hallmark of dystrophinopathies is the persistent relative sparing of the sartorius and gracilis (and often the semitendinosus in *BMD*) [13]. In contrast, our patient exhibited early hyperintense streaking in these muscles by age 13, culminating in end-stage, complete fatty replacement (Mercuri grade 4) by age 16. Furthermore, while the quadriceps muscles typically undergo progressive and often severe fatty degeneration in *DMD/BMD* [7,13], the anterior compartment in our patient, particularly the rectus femoris and vastus lateralis, remained relatively preserved and maintained near-normal muscle volume at 16 years of age. This advanced-stage “posterior-to-anterior gradient” acts as a robust differentiator from *DND/BMD*.

### 3.2. Comparison with Other LGMD Subtypes

The present case also demonstrates crucial imaging divergences when compared to other prevalent LGMD subtypes. While early involvement of the posterior thigh is shared with LGMD R1 (calpainopathy), R2 (dysferlinopathy), and R9 (FKRP-related dystrophy) [3,14], the subsequent progression pattern in our patient is highly atypical. In subtypes such as LGMD R1, R2, R3–R6 (sarcoglycanopathies), and R9, the sartorius and gracilis are characteristically spared until the very late stages, while the vasti muscles (particularly the vastus lateralis and intermedius) undergo early progressive fatty replacement [3,14]. The inverse pattern observed in our patient, characterized by advanced degeneration of the sartorius and gracilis together with sustained preservation of both the rectus femoris and vastus lateralis, represents a distinctive imaging feature that may aid in the differential diagnosis of LGMD R18.

### 3.3. Comparison with Previously Reported LGMD R18

When evaluated against the existing LGMD R18 literature, our case highlights significant phenotypic expansions. A recognized core imaging signature of this disease is the severe degeneration of the sartorius juxtaposed with the relative preservation of the vastus lateralis [15]. Our findings align with this specific parameter. However, our longitudinal data reveals a significant spatiotemporal departure from prior reports. While previous cross-sectional studies have reported a predilection for anterior and medial muscle group involvement—typically citing the rectus femoris as one of the most severely affected muscles despite adjacent vastus lateralis sparing [15]—our patient exhibited entirely different compartment dynamics. Fatty replacement was heavily concentrated in the posterior and medial compartments, while the anterior compartment, particularly the rectus femoris, exhibited persistent, robust preservation through the advanced stages.

## 4. Conclusions

We report a patient with LGMD R18 who initially presented with a low ceruloplasmin level and underwent six years of longitudinal MRI follow-up. The diagnosis was established through the integration of clinical examination, longitudinal neurological follow-up, laboratory findings, electromyography, genetic testing, and muscle MRI evaluation. The six-year serial MRI follow-up presented in this case provides a detailed temporal characterization of muscle involvement that cannot be obtained from cross-sectional observations alone, offering novel insights into both the clinical presentation and radiological progression of this condition. These findings highlight the value of longitudinal MRI in delineating the dynamic evolution of muscle degeneration. Such data may also contribute to future research aimed at developing imaging biomarkers and improving disease monitoring in inherited myopathies. In addition, longitudinal imaging data, in conjunction with functional assessments of daily living, may inform future research on assistive technologies, including artificial intelligence (AI)-assisted rehabilitation approaches and robotic exoskeleton systems, by enabling more precise characterization of muscle involvement patterns.

## Figures and Tables

**Table 1:** Chronological clinical, biochemical, and radiological timeline of the patient.

**Age 9: INITIAL PRESENTATION**	
Incidental detection of elevated liver enzymes. Lab Workup: Persistently elevated transaminases, low ceruloplasmin, and high CK.	
**Ages 9: DIFFERENTIAL DIAGNOSIS WORKUP**	
Suspected Wilson’s Disease (WD)	EXCLUDED VIA:
	- Slit-lamp: No Kayser–Fleischer rings. - Brain MRI: No basal ganglia/brainstem lesions. - Genetics: Negative for ATP7B mutations.
Suspected Neuromuscular Disorder	CONFIRMED VIA:
	- Needle EMG: Primary myopathic process. - NGS: Homozygous TRAPPC11 variant (c.2938G>A, p.G980R). DEFINITIVE DIAGNOSIS: LGMD R18.
**Ages 11–16: 6-YEAR LONGITUDINAL SURVEILLANCE**	
Biochemical Monitoring (Fluctuating but persistent anomalies):	
	- Serum CK: Markedly elevated (Mean: 7429 ± 1615 IU/L). - Ceruloplasmin: Persistently low (Mean: 10.1 ± 1.3 mg/dL).
Serial Thigh Muscle MRI Evolution (“Posterior-to-Anterior Gradient”):	
	- Age 11: Early fatty infiltration limited to AM and ST muscles. - Age 12: Progression in posterior compartment; anterior & medial muscles spared. - Age 13: Advanced posterior degeneration; early hyperintense streaking in S and G. - Age 16: End-stage fatty replacement (Mercuri Grade 4) of posterior/medial compartments; Marked relative preservation of the anterior compartment (RF and VL).
**Age 16: CURRENT OUTCOME & STATUS**	
	Interventions: Conservative management and functional follow-up. Functional Status: Independent in ADLs, active social/school life; progressive fatigue during stair climbing due to proximal weakness.

**Table 2:** Differential diagnostic matrix summarizing the distinct and overlapping clinical, biochemical, and radiological features between LGMD R18 and Wilson’s disease.

Disease	Clinical & Laboratory Features
Typical LGMD R18 (*TRAPPC11*-related)	Neuromuscular Manifestations: Progressive skeletal muscle weakness (proximal/limb-girdle distribution), muscle pain and atrophy. Markedly Elevated Creatine Kinase (CK): Persistently significant high serum CK levels. Muscle Biopsy Findings: Dystrophic or myopathic changes. Distinct Muscle MRI Signatures: Selective progressive fatty infiltration showing a “posterior-to-anterior gradient” in thigh muscles. Systemic/CNS Anomalies: Microcephaly, short stature, cataracts, seizures, or structural brain abnormalities (such as cerebral atrophy or corpus callosum hypoplasia).
Shared/Overlapping Features (Diagnostic Mimicry)	Hepatic Involvement/Dysfunction: Both conditions can feature liver disease, presenting with hepatomegaly, hepatic steatosis, or liver fibrosis. Elevated Serum Transaminases: Elevated liver enzymes (AST/ALT) are frequently found in both disorders, which can initially mislead clinicians toward a primary hepatic cause. Abnormal Low Ceruloplasmin: Decreased serum ceruloplasmin levels can occur in LGMD R18 patients, a classic laboratory hallmark highly characteristic of Wilson disease. Neurological Involvement: Both can manifest with neurological or motor-related disabilities.
Typical Wilson Disease (Absent in LGMD R18)	Ocular Copper Deposition: Presence of Kayser–Fleischer (K-F) rings on slit-lamp eye examination. Genetic Diagnosis: Pathogenic mutations strictly involving the *ATP7B* gene (which functions in copper transport) rather than *TRAPPC11*. Systemic Copper Overload: Biochemical signatures showing abnormal copper metabolism, such as elevated 24-h urinary copper excretion or high hepatic copper concentration (not linked to global glycosylation or vesicle transport failure).

## Data Availability

The original contributions presented in this study are included in the article. Further inquiries can be directed to the corresponding author.

## Abbreviations

The following abbreviations are used in this manuscript:

LGMDs	Limb-girdle muscular dystrophies
TRAPP	trafficking protein particle
MRI	magnetic resonance imaging
DMS	Duchenne muscular dystrophy
BMD	Becker muscular dystrophy
CK	creatine kinase
